# A Case of Testicular Malakoplakia With Markedly High Signal Intensity on Fat‐Suppressed T1‐Weighted Images

**DOI:** 10.1002/iju5.70187

**Published:** 2026-04-27

**Authors:** Fumiko Yagi, Hirotaka Akita, Takeo Kosaka, Akihisa Ueno, Teppei Kotera, Masato Kobayashi, Hideaki Mekada, Mototsugu Oya, Masahiro Jinzaki

**Affiliations:** ^1^ Department of Radiology Keio University School of Medicine Tokyo Japan; ^2^ Department of Urology Keio University School of Medicine Tokyo Japan; ^3^ Division of Diagnostic Pathology Keio University Hospital Tokyo Japan

**Keywords:** differential diagnosis, magnetic resonance imaging, malakoplakia, T1‐weighted imaging, testis

## Abstract

**Introduction:**

Testicular malakoplakia is a very rare, chronic granulomatous inflammatory condition that frequently resembles testicular malignancy on imaging studies. Here, we report a case of testicular malakoplakia with characteristic magnetic resonance imaging (MRI) findings and present a brief review of the literature.

**Case Presentation:**

A man in his 70s presented with left‐sided scrotal pain. Ultrasonography revealed a well‐defined hypoechoic intratesticular mass without internal vascularity, whereas computed tomography revealed enlargement of the left testis with a relative hyperattenuation area. MRI demonstrated low signal intensity on T2‐weighted images, diffusion restriction, and a markedly high signal intensity on fat‐suppressed T1‐weighted images with contrast enhancement. Based on these findings, a malignant testicular tumor was suspected, and radical orchidectomy was conducted. Histopathological examination confirmed testicular malakoplakia with characteristic Michaelis–Gutmann bodies.

**Conclusion:**

This case highlights the importance of recognizing T1 shortening on fat‐suppressed T1‐weighted MRI as a potential imaging modality for malakoplakia.

## Keynote Message

Testicular malakoplakia is a rare inflammatory condition that can mimic testicular cancer. Marked T1 shortening on fat‐suppressed T1‐weighted MRI may reflect calcium and iron deposition within Michaelis–Gutmann bodies and aid diagnosis.

## 1 Introduction

Malakoplakia is a rare chronic inflammatory disorder caused by defective macrophage phagocytic activity, usually associated with bacterial infection. It primarily involves the genitourinary tract, particularly the urinary bladder, whereas testicular involvement is rare. Because of its mass‐like appearance, it is often misdiagnosed as malignancy. We report a rare case of testicular malakoplakia with characteristic magnetic resonance imaging (MRI) findings and discuss its imaging features and differential diagnosis.

## 2 Case Presentation

**FIGURE 1:**
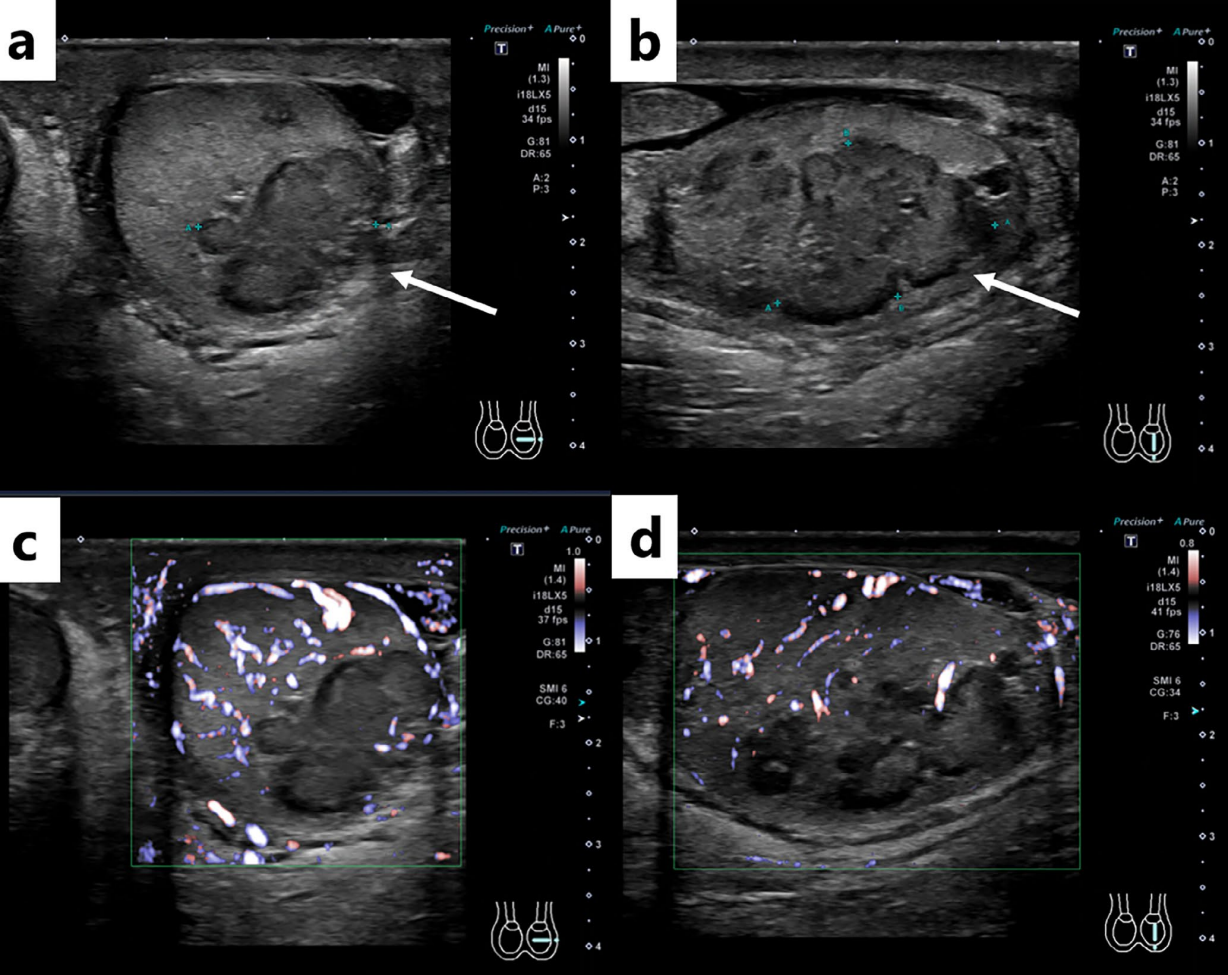
Ultrasonographic findings of the left testis. Ultrasonography reveals a mass in the left testis appearing as multiple fused nodules (a, b; arrow). The borders are well‐defined, the margins are hypoechoic, and no detectable blood flow is noted (c, d).

**FIGURE 2:**
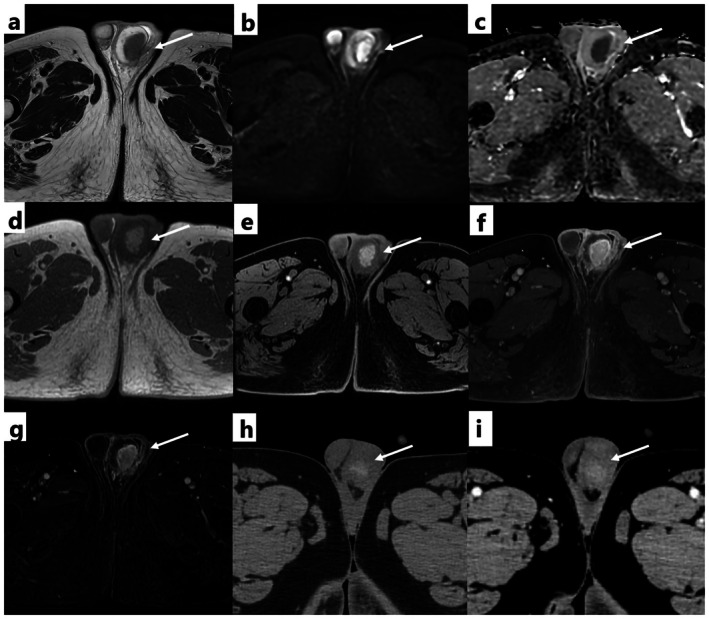
Magnetic resonance imaging (MRI) and computed tomography (CT) findings of the left testis. (a) T2‐weighed image, (b) diffusion‐weighted image (*b* = 1000 s/mm^2^), (c) ADC map, (d) T1‐weighed image, (e) fat‐suppressed T1‐weighed image, (f) contrast‐enhanced MRI (g) subtraction image (before and after contrast enhancement), (h) non‐contrast CT. (i) Contrast‐enhanced CT. MRI shows low signal intensity in the left testicular mass on T2WI (a; arrow), with diffusion restriction on diffusion‐weighted imaging (b; arrow) and the ADC map (c; arrow) with an ADC value of 0.84 × 10^−3^ mm^2^/s. T1WI (d; arrow) and fsT1WI (e; arrow) show high signal intensity and contrast enhancement is seen (d, e, f, and g; arrow). Non‐contrast CT (h) shows an enlarged left testis with a hyperattenuation area (arrow). Contrast‐enhanced CT (i) demonstrates only faint and indistinct enhancement of the lesion (arrow).

**FIGURE 3:**
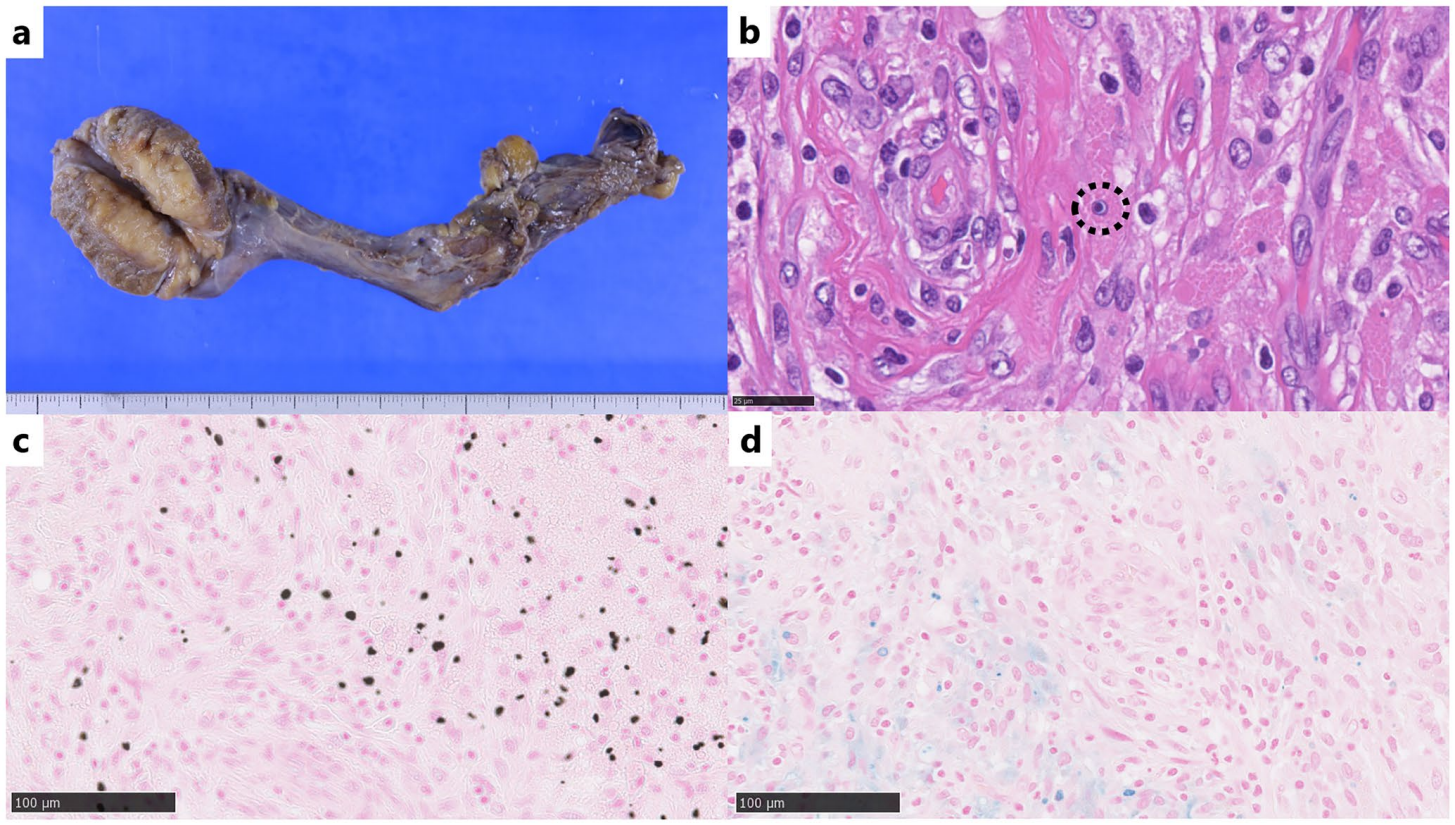
Pathological findings. (a) Gross pathological examination, (b) Hematoxylin and Eosin staining, (c) von Kossa staining, (d) Berlin blue staining. Gross pathological examination revealed an enlarged testis and a 3.4‐cm‐sized yellow and white lesion in the left testis (a). Infiltration of spindled shaped von Hansemann cells with lymphocytes and collagen fibers and Michaelis–Gutmann bodies (b; dotted circle) is observed in the cytoplasm. The von Kossa staining (c) for calcium and Berlin blue staining (d) for iron highlight Michaelis–Gutmann bodies.

A man in his 70s presented with left scrotal pain that had persisted for several months. He was referred to our hospital with prostate cancer and pelvic lymph node metastasis. Biopsy showed ductal adenocarcinoma (cT3a), with an initial PSA level of 18.203 ng/mL, which decreased to 2.240 ng/mL after subcutaneous degarelix. His medical history was notable for hypertension, dyslipidemia, and hyperuricemia. There was no relevant family history. He smoked approximately 20 cigarettes daily from age 20 to 68 years and did not consume alcohol. At presentation, he had a fever (38.1°C) and a firm, enlarged left testis. Laboratory test results revealed leukocytosis (white blood cell count 15.6 × 10^3^/μL) and elevated inflammatory markers (C‐reactive protein 18.04 mg/dL). The serum lactate dehydrogenase level was 203 U/L. Tumor markers, including human chorionic gonadotropin (< 0.3 mIU/mL) and α‐fetoprotein (2 ng/mL), were within normal limits. Soluble interleukin‐2 receptor levels were mildly elevated (684 U/mL). Urinalysis revealed pyuria, bacteriuria, and microscopic hematuria. Urine culture grew *Escherichia coli* and a small amount of *Enterococcus faecalis* . Scrotal ultrasonography demonstrated a mass in the left testis consisting of multiple coalescent nodules. The lesion was well defined with hypoechoic margins and demonstrated no detectable internal blood flow on color Doppler imaging (Figure 1). Computed tomography (CT) showed enlargement of the left testis with an area of relatively increased attenuation compared with the contralateral side. Contrast‐enhanced CT showed very faint, indistinct enhancement of the left testicular lesion (Figure 2). He was treated with oral levofloxacin (500 mg/day) for 14 days, which improved the fever and pain; however, the testicular mass and enlargement persisted. MRI was therefore performed 24 days after presentation. MRI showed a left testicular mass with low signal intensity on T2‐weighted images and diffusion restriction on diffusion‐weighted images. On fat‐suppressed T1‐weighted images, the lesion showed markedly high signal intensity. Contrast‐enhanced MRI revealed enhancement of the lesion (Figure 2). These findings raised suspicion of a malignant testicular tumor. Considering the imaging findings and clinical background, metastatic disease and malignant melanoma were included in the differential diagnosis. Therefore, a radical left orchidectomy was conducted 42 days after presentation. Gross pathological examination showed an enlarged testis and a 3.4‐cm‐sized yellow and white lesion was detected in the left testis (Figure 3). Microscopically, Michaelis–Gutmann bodies were confirmed by hematoxylin and eosin staining, and they were positive for von Kossa staining and Berlin blue staining (Figure 3). The final pathological diagnosis was testicular malakoplakia. Testicular pain and enlargement improved after surgery. C‐reactive protein levels decreased to 1.31 mg/dL 10 days after starting antibiotics and further to 0.09 mg/dL postoperatively.

## 3 Discussion

Malakoplakia is a rare, chronic inflammatory condition caused by defective macrophage phagocytic function in response to bacterial infection, most commonly *E. coli* [1]. It is typically preceded by infectious cystitis and occurs most often in immunocompromised individuals including older patients and those with diabetes mellitus, *human immunodeficiency virus* infection, organ transplantation, or malignancy [2, 3]. The reported age range is wide, from infancy to advanced age [3], with a male predominance (male‐to‐female ratio approximately 4:1) [2]. Within the urinary tract, malakoplakia most frequently involves the urinary bladder, whereas testicular involvement is rare [4]. In addition to the genitourinary system, malakoplakia has been described in numerous organs including the gastrointestinal tract, bones, lungs, lymph nodes, and skin [3]. Treatment usually involves prolonged antibiotic therapy, with surgical resection considered for selected cases [2].

Histopathologically, malakoplakia is characterized by infiltration of enlarged foamy histiocytes with eccentric, hyperchromatic nuclei and eosinophilic granular cytoplasm, called von Hansemann cells, which are often arranged in sheets, with a variably mixed population of neutrophils, lymphocytes, and plasma cells [3]. Another defining feature is the presence of Michaelis–Gutmann bodies, which are intracytoplasmic laminated concretions formed by calcified, iron‐containing phagolysosomes [3]. These inclusions typically show positivity on special stains, including periodic acid–Schiff, Perls' Prussian blue (Berlin blue), and von Kossa stains, and are critical for a definitive diagnosis.

Testicular malakoplakia is typically unilateral, with isolated testicular involvement occurring more than combined involvement of the testis and epididymis [5, 6, 7]. Patients most often present with scrotal pain and testicular enlargement [7]. On imaging, testicular malakoplakia usually appears as a mass‐like enlargement and may demonstrate heterogeneous internal architecture with cystic components [4, 8]. In some cases, complications such as abscess formation or thrombosis have been reported [9], making an accurate diagnosis more difficult. Imaging findings of malakoplakia vary widely in both morphology and signal intensity [10]. Previous MRI studies of prostatic malakoplakia [11] have described lesions with high signal intensity on T1‐weighted images, indicating that calcium and iron deposition within Michaelis–Gutmann bodies may cause T1 shortening. In the present case, the testicular lesion demonstrated a markedly high signal intensity on fat‐suppressed T1‐weighted images, supporting a similar underlying mechanism and underscoring this feature as a potentially important diagnostic clue.

From a radiological perspective, several entities can show T1 shortening. Fat‐containing lesions were excluded on fat‐suppressed T1‐weighted imaging, whereas contrast‐enhanced imaging helps distinguish hemorrhage from solid tumors. Although enhancement on contrast‐enhanced CT was subtle, even faint true enhancement supported a solid inflammatory process rather than an intratesticular hematoma, which typically shows no enhancement. Malignant melanoma can show intrinsic T1 hyperintensity due to the paramagnetic properties of melanin and may be suspected when a lesion appears black on gross inspection or when there is a history of melanoma [12]. Tuberculosis is another important differential diagnosis, since T1 shortening may be caused by paramagnetic substances, such as macrophage‐laden oxygen‐free radicals, within granulomatous inflammation [13, 14, 15, 16].

Malakoplakia may mimic malignancy because of its mass‐like appearance and possible involvement of surrounding structures [3, 4, 7, 10]. Histologically, Michaelis–Gutmann bodies may be misinterpreted as nucleoli, leading to confusion with urothelial carcinoma or malignant lymphoma; therefore, immunohistochemical staining is important for accurate diagnosis. When malakoplakia is suspected on imaging, communication with pathologists is essential to ensure appropriate pathological evaluation.

## 4 Conclusion

Testicular malakoplakia is a rare condition that can mimic testicular malignancy on imaging. Marked T1 shortening on fat‐suppressed T1‐weighted MRI may provide an important diagnostic clue. Awareness of this entity may help avoid diagnostic pitfalls.

## Ethics Statement

The authors have nothing to report.

## Consent

The authors have nothing to report.

## Conflicts of Interest

Mototsugu Oya is an Editorial Board member of the International Journal of Urology and a co‐author of this article but was not involved in its editorial decision‐making. The other authors declare no conflicts of interest.

## Data Availability

The authors have nothing to report.
